# Decentralized clinical trials in the trial innovation network: Value, strategies, and lessons learned

**DOI:** 10.1017/cts.2023.597

**Published:** 2023-07-25

**Authors:** Daniel F. Hanley, Gordon R. Bernard, Consuelo H. Wilkins, Harry P. Selker, Jamie P. Dwyer, J. Michael Dean, Daniel Kelly Benjamin, Sarah E. Dunsmore, Salina P. Waddy, Kenneth L. Wiley, Marisha E. Palm, W. Andrew Mould, Daniel F. Ford, Jeri S. Burr, Jacqueline Huvane, Karen Lane, Lori Poole, Terri L. Edwards, Nan Kennedy, Leslie R. Boone, Jasmine Bell, Emily Serdoz, Loretta M. Byrne, Paul A. Harris

**Affiliations:** 1 Johns Hopkins University School of Medicine, Baltimore, MD, USA; 2 Johns Hopkins Institute for Clinical and Translational Research, Baltimore, MD, USA; 3 Department of Medicine, Vanderbilt University Medical Center, Nashville, TN, USA; 4 Vanderbilt Institute for Clinical and Translational Research, Nashville, TN, USA; 5 Department of Internal Medicine, Meharry Medical College, Nashville, TN, USA; 6 Department of Medicine, Tufts University, Boston, MA, USA; 7 Tufts Clinical and Translational Science Institute, Tufts University, Boston, MA, USA; 8 Institute for Clinical Research and Health Policy Studies, Tufts Medical Center, Boston, MA, USA; 9 University of Utah Health, Salt Lake City, UT, USA; 10 Utah Clinical and Translational Sciences Institute, Salt Lake City, UT, USA; 11 Duke University School of Medicine, Durham, NC, USA; 12 Duke Clinical Research Institute, Durham, NC, USA; 13 National Center for Advancing Translational Sciences, Bethesda, MD, USA; 14 Johns Hopkins BIOS Clinical Trials Coordinating Center, Baltimore, MD, USA; 15 Department of Biomedical Informatics, Vanderbilt University Medical Center, Nashville, TN, USA

**Keywords:** Decentralized trials, hybrid trials, CTSA, trial innovation network, inclusive recruitment, remote trials, remote technology, rural recruitment, remote recruitment, remote intervention, remote data collection, MyCap, remote trial monitoring

## Abstract

New technologies and disruptions related to Coronavirus disease-2019 have led to expansion of decentralized approaches to clinical trials. Remote tools and methods hold promise for increasing trial efficiency and reducing burdens and barriers by facilitating participation outside of traditional clinical settings and taking studies directly to participants. The Trial Innovation Network, established in 2016 by the National Center for Advancing Clinical and Translational Science to address critical roadblocks in clinical research and accelerate the translational research process, has consulted on over 400 research study proposals to date. Its recommendations for decentralized approaches have included eConsent, participant-informed study design, remote intervention, study task reminders, social media recruitment, and return of results for participants. Some clinical trial elements have worked well when decentralized, while others, including remote recruitment and patient monitoring, need further refinement and assessment to determine their value. Partially decentralized, or “hybrid” trials, offer a first step to optimizing remote methods. Decentralized processes demonstrate potential to improve urban-rural diversity, but their impact on inclusion of racially and ethnically marginalized populations requires further study. To optimize inclusive participation in decentralized clinical trials, efforts must be made to build trust among marginalized communities, and to ensure access to remote technology.

## Introduction

The advent of new digital technologies has expanded the ability of researchers to reach participants directly, outside of traditional healthcare channels [1]. The onset of the SARS-CoV-2 (COVID-19) pandemic in 2020 increased trial consultations conducted by the Trial Innovation Network (TIN) [2] and provided motivation to establish remote trial processes and minimize disease transmission. A remote or “decentralized” trial has been defined as: “A clinical trial utilizing technology, processes, and/or services that create the opportunity to reduce or eliminate the need for participants to physically visit a traditional research site [3].” “Hybrid” trials, incorporating some elements of decentralization driven by study needs, individual site capabilities, and patient preferences have become more common [4–10]. A recent study demonstrated that implementing decentralized approaches can significantly improve trial efficiency and lower costs by reducing screen failure rates and making consent and enrollment more convenient; easing the burden of time and travel by providing interventions remotely; facilitating remote measurement of outcomes; and increasing trial speed by minimizing protocol amendments [11]. Moreover, adopting processes that bring research more directly to the participant can make clinical trials more participant-centered [12].

The TIN was established in 2016 by the National Center for Advancing Clinical and Translational Science (NCATS) to increase the efficiency and effectiveness of clinical trials by embedding innovation and critical evaluation into the clinical trials process. The TIN consists of three Trial Innovation Centers (TICs), one Recruitment Innovation Center (RIC) [13], and a collaborating network of over 60 Clinical and Translational Science Award (CTSA) program institutions. The TIN has consulted with research teams from across the nation on over 400 clinical trials. Although the experience of the TIN with completely decentralized clinical trials remains limited, the TICs and RIC have increasingly considered aspects of decentralized study design for a wide variety of clinical trials over the past six years.

In this paper, we review the use, benefits, and opportunities afforded by decentralization, and describe examples of how trials by TIN investigators have used remote methods to increase multicenter trial efficiency, reduce participant burden, and achieve a broader reach of participation. We also highlight some of the challenges associated with decentralized trial design and areas of uncertainty.

## TIN Decentralized Trials – Case Studies

Decentralized and hybrid trials can include many different technologies and approaches for enabling remote recruitment and participation. These approaches should be designed to reduce participant burden while maintaining efficient and high-quality data collection and appropriate safety monitoring. We highlight two trials below as case studies to illustrate TIN efforts that incorporated decentralized processes into complex protocols.

### **R**hythm **E**valuation for **A**nti**C**oagula**T**ion with Continuous Monitoring of **A**trial **F**ibrillation (REACT-AF)

REACT-AF [14] is a large pragmatic study of over 5000 participants that compares rates of ischemic stroke, systemic embolization, major bleeding, and death in patients receiving chronic novel oral anticoagulation (NOAC) therapy for atrial fibrillation (AF) versus those receiving time-delimited, targeted NOAC therapy. The REACT-AF consultation with one TIC led to changes in study design expected to enhance data collection, reduce site burden, and decrease trial costs by 50%, mainly by instituting decentralized methods. A central element of this hybrid trial is the use of a remote wearable – an Apple Watch – to sense AF in participants. Using a direct-to-participant approach, the clinical trial mobile platform Eureka pairs with the watch and allows direct messaging and electronic patient-reported outcomes (ePRO) data collection in place of traditional follow-up visits [15]. Once in-person enrollment is complete, no additional in-person study visits are required, eliminating the burden of participant travel, widening the geographic reach of the intervention, reducing study team effort, and decreasing the overall budget of the trial. Site and central trial oversight of participant use of the device (watch) is a major organizational activity directed at oversight of data quality and participant adherence. The REACT-AF trial is currently in the startup phase and is activating the first wave of 40 sites with enrollment of first participant in July 2023.

### **PR**agmatic **EV**aluation of Ev**ENT**s **A**nd **B**enefits of **L**ipid-Lowering in Old**E**r Adults (PREVENTABLE)

PREVENTABLE [16], another example of a hybrid trial, is a study of 15,000 community-dwelling adults aged 75+ designed to evaluate whether a statin prevents the onset of dementia. This pragmatic trial aims to include adults from racial and ethnic minority groups who live in diverse geographic locations. The RIC developed a process to identify barriers to study participation and assess the protocol activities by engaging community experts who matched the eligibility criteria of the PREVENTABLE study. This was accomplished through ResearchMatch [17], an online platform of about 145,000 volunteers that connects potential trial participants with researchers at medical centers nationwide. The survey asked volunteers for their perceptions about information needs, research benefits, reasons for declining participation, home study visits, computer access, transportation needs, and who would influence their decision to participate in a clinical trial. Key barriers to potential trial participation included transportation to study visits, difficulty understanding the consent form, and access to study medication. In addition, eight community experts aged 75+ years reviewed the protocol and shared concerns and solutions regarding reaching older adults living in rural areas; being inclusive of those living in communal settings such as assisted living centers; using eConsent [18] or video consent to aid with understanding and translation of study participation requirements; providing convenient access to study medication; and finding solutions to lack of patient transportation. Consequently, the study team implemented remote eConsent using a tablet or smartphone and provided consent videos, enrollment via telehealth, home delivery of study medication, and study follow-up via patient electronic health record (EHR) review, telephone, or home visits. In addition to community input into the study design, the study team convened a virtual Participant Advisory Group to provide ongoing input on protocols, procedures, communications, recruitment and retention strategies, and return of value ideas. The diversity of the Participant Advisory Group aligns with the demographics of the PREVENTABLE participant population.

The PREVENTABLE trial does require inpatient visits for blood draws and performance tests; however, visits are minimized using a call center for annual assessments, which include cognitive function and memory tests. Some monitoring data and endpoints are also collected remotely, using the EHR, National Death Index, and Medicare databases to query for participant events. Recruitment for the 5-year study began in September 2020. To date, the study has 96 sites activated and 5,286 participants enrolled.

## Decentralized Trials Processes and TIN Examples

**Figure 1.**
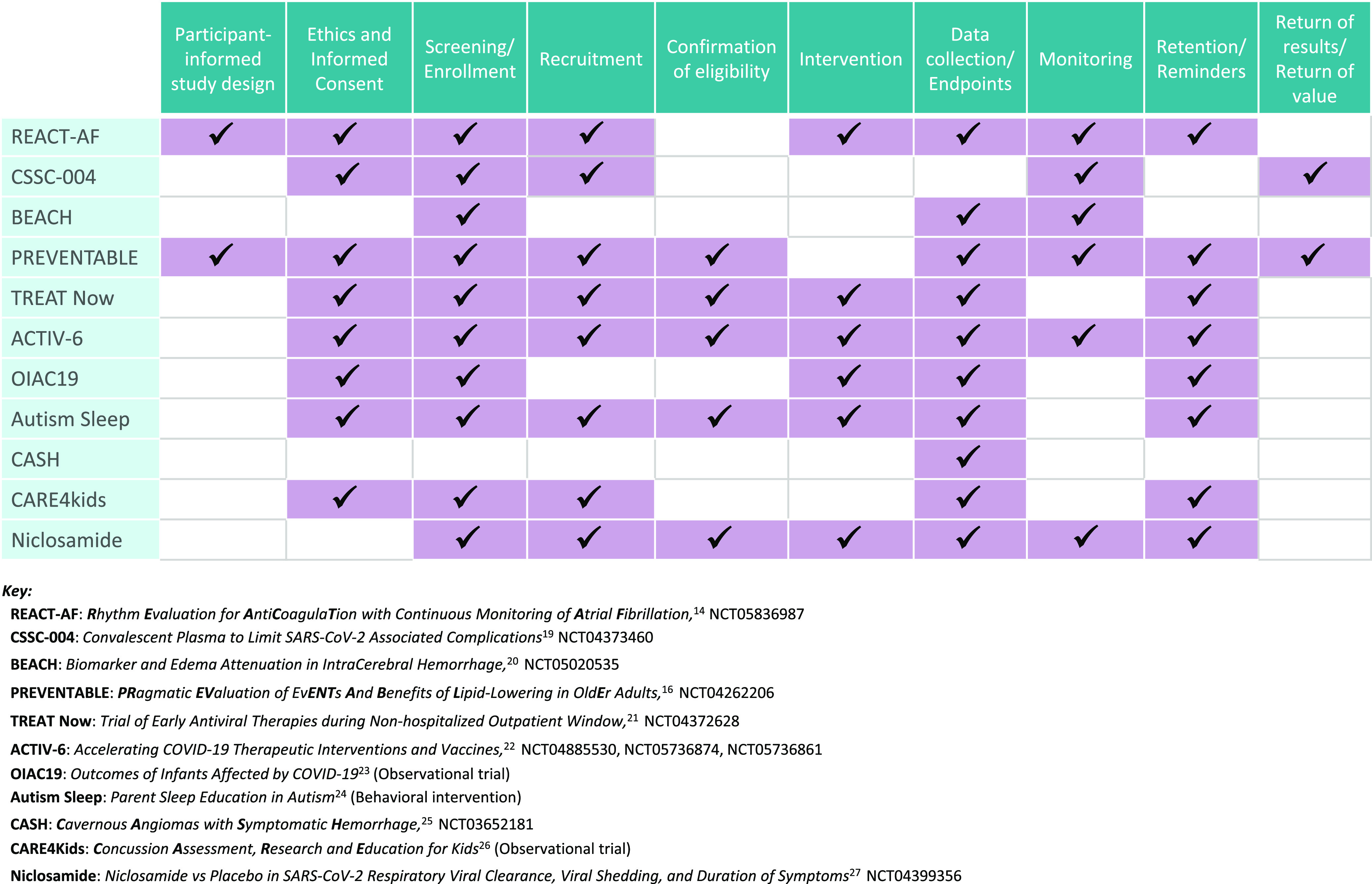
Decentralized elements of design for trials conducted by Trial Innovation Center (TIC) or Recruitment Innovation (RIC) Center investigators or through TIC or RIC coordinating centers.

Fig. 1 displays the flow of a clinical trial, from design through retention, listed as steps that can potentially be executed remotely. The column on the left displays 11 trials [14,16,19–27] supported and/or conducted by TIC/RIC investigators that employ one or more decentralized elements, some of which will be discussed in the text. Notably, none of the trials listed are fully decentralized; instead, they employ different combinations of decentralized elements in a fit-for-purpose approach.

### Participant-informed Study Design

Input on study design from stakeholder community members can help investigators design studies that have increased impact and decreased participant burden. Input can be obtained through teleconference sessions, remotely held Community Engagement Studios [28], or online surveys. For several TIN studies, we leveraged the “rapid community feedback” feature of ResearchMatch [17], which can deploy opinion polls to help researchers gain insight from a diverse population on study design, recruitment messaging, preferences for returning research findings, and more. In the PREVENTABLE case study mentioned above, we polled 2,607 ResearchMatch volunteers and tallied 107 rapid responses. For the REACT-AF study, we used an online survey to query 46 ResearchMatch volunteers meeting study eligibility criteria to ascertain their willingness to use Apple Watch wearable technology. Responses demonstrated that this requirement was feasible.

We have not been made aware of any drawbacks from the activities that involve seeking participant feedback. The study team can decide not to follow the advice of the participants, but at least the information has been shared.

### Ethics and Informed Consent

IRB approval can be supported with decentralized methods by using an electronic portal for IRB reliance and subsequent approvals. Decentralized methods can also be used to support informed participant consent, including eConsent via phone or video call, and signature via DocuSign, email, or REDCap [18]. This informed consent (IC) process can be synchronous or asynchronous, and incorporate interactive or multimedia components [30]. Since remote IC relies heavily on technology platforms, such as video conferencing or electronic signatures, drawbacks could include technical issues such as poor Internet connectivity, software glitches, or hardware malfunctions, which can disrupt the process and hinder effective communication. In addition, privacy and security need extra attention since remote IC requires the transmission of sensitive personal information over digital channels. Lack of face-to-face conversation during the consent process may contribute to telehealth fatigue, limited non-verbal cues, and inadequate ability to ask questions or seek clarification, which could potentially compromise the participant’s understanding and informed decision-making.

### Screening

Determination of participant eligibility can be accomplished remotely through EHR assessment, online surveys, or by leveraging technological advancements in algorithm development and machine learning to automate aspects of screening. The Biomarker and Edema Attenuation in IntraCerebral Hemorrhage (BEACH) trial [20] includes an AI algorithm deployed at each trial site that automatically reads every head computerized tomography (CT) scan taken, identifies and measures hemorrhages, and transmits this information to research staff in real time via a mobile application. This streamlines the identification of potential trial candidates and allows research staff at trial sites to conduct clinical screening and consenting more quickly. Even if all inclusion/exclusion criteria cannot be verified independent of a traditional clinic visit, efficiencies can still be created in the screening process by leveraging electronic platforms, direct-to-participant methods, and technology to increase saturation of likely participants. The Convalescent Plasma to Limit SARS-CoV-2 Associated Complications (CSSC-004) trial [19] employed direct transfers of data from EHRs to trial case report forms instead of collecting data at the clinical trial site.

It should be noted that remote screening may limit the completeness of screening data or evaluation of potential participant attitudes toward a study. For example, a head CT scan that automatically measures the hemorrhage to be outside of the acceptable range or an EHR pull that captures an exclusion may result in the study coordinator never advancing to the clinic visit with the patient to assess the remaining inclusion/exclusion criteria or to determine potential participant interest in the study.

### Recruitment

Remote recruitment methods include EHR patient portals, social media, TV/radio announcements, and online ads. In the PREVENTABLE [16] trial, potential participants were identified by creating a phenotype derived from the inclusion and exclusion criteria. Recruitment strategies included brochures, flyers, postcards, self-mailers, social media, and teleconference meetings for interested participants. Informational videos provided on websites or via social media explain the study’s conduct and requirements. The Outcomes of Infants Affected by COVID-19 (OIAC19) [23] trial identified eligible mothers who were sent a REDCap[31] link through which they were able to enroll themselves and their infants. While decentralized methods for inviting recruitment into clinical trials may allow researchers to reach wider audiences and tailor recruitment messages, these approaches may unintentionally exclude individuals with limited access to technologies including Internet and EHR patient portals. Future considerations should include diverse recruitment methods that do not depend wholly on technology.

### Confirmation of Eligibility

Confirmation of eligibility to participate in a research study can be approached with a wide array of remote tools and methods, including online survey responses, phone interviews, video calls, electronic source (eSource) data entered directly into electronic case report forms (eCRFs), physiologic data (e.g., blood pressure, heart rate), pupillometer devices, magnetic resonance imaging (MRIs), EHR data, lab results, and digital biomarkers. An example of remote initial evaluation is the Trial of Early Antiviral Therapies during Non-hospitalized Outpatient Window (TREAT Now) study [21]. Researchers conducted a Google Ads campaign, in which individuals diagnosed with COVID-19 could click on an ad for a survey to determine study eligibility. Those who qualified were redirected to a study website to continue the consenting and enrollment processes. A study coordinator called the participant to confirm eligibility and answer questions. Participants were provided with a link to upload their positive COVID-19 test if not already accessible in the local EHR. One concern in relying on remote evaluation would be the accuracy or even truthfulness of the data. In-person examination and history taking are likely to be taken more seriously.

### Intervention

The remote application of a treatment or procedure, administration of a drug, or use of a medical device are examples of decentralized interventions. These can be delivered to participants via direct-to-patient shipping, such as medications being delivered to participants in the TREAT Now [21] study; the “Clinical-trial-in-a-box” sent to participants in the Niclosamide vs Placebo in SARS-CoV-2 Respiratory Viral Clearance, Viral Shedding, and Duration of Symptoms (Niclosamide) trial [27]; home visits by study staff; teleconferencing; and platforms such as MyCap [32], REDCap, or patient portals. In the REACT-AF [14] study, an Apple Watch is provided to the participant pre-loaded with a cloud-based mobile application, Eureka, which notifies the participant of an AF event and subsequently instructs them when to start and when to stop taking their medication.[15] Follow-up data are collected via the mobile application; participants are able to carry out the remainder of the study from home.

A possible disadvantage of these remote methods is that they remove some data acquisition responsibility from paid research professionals at sites and place it into the hands of participants, who may have less understanding of data quality, completeness, and integrity. Because of this concern, significant effort is required to ensure instructions to participants are clear and obvious. Moreover, depending on the system and circumstances, resource levels for technical operational support staff may need to be higher given the need to support end-users with varying levels of technical expertise or training.

### Data Collection

**Figure 2.**
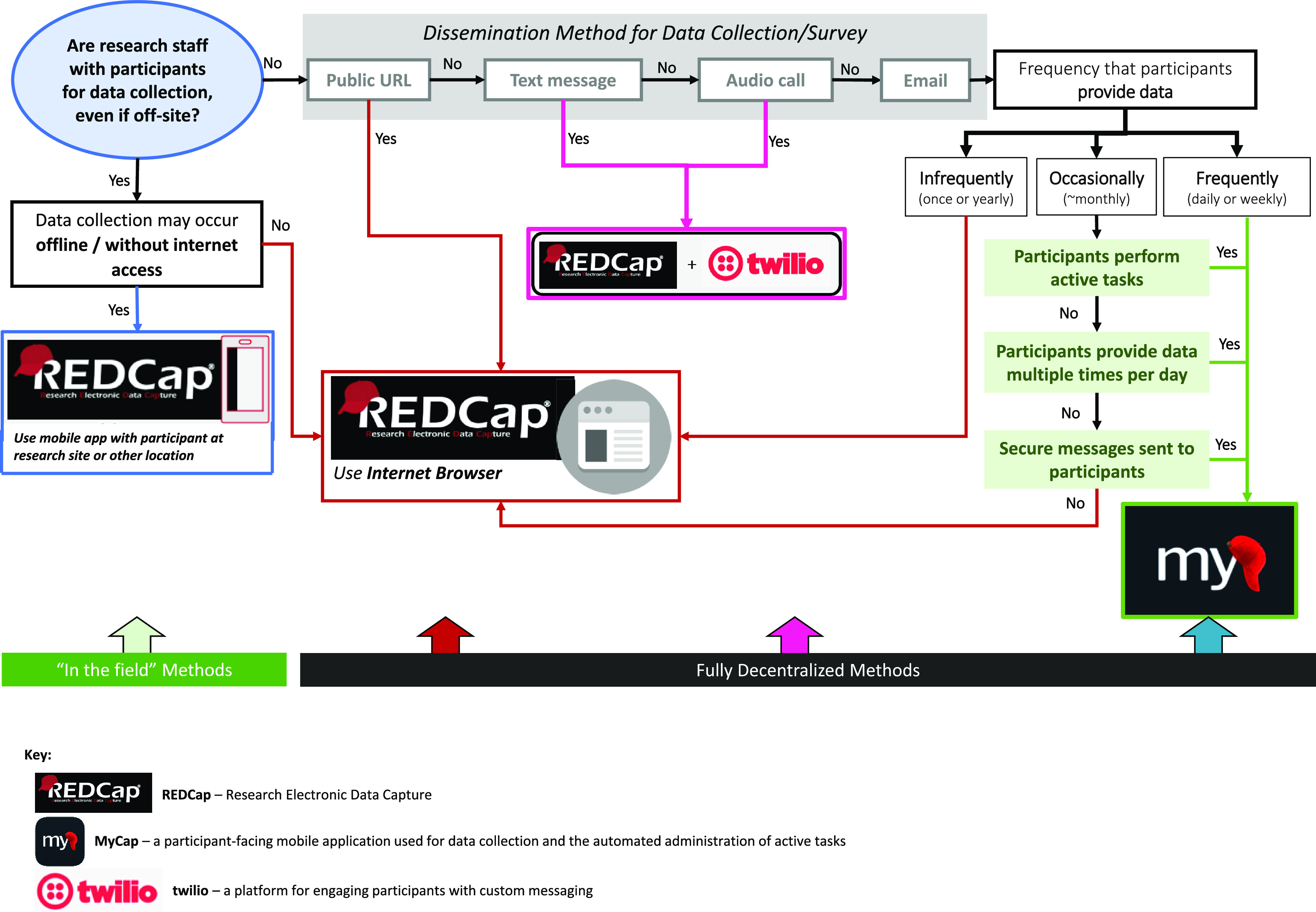
Decentralized and hybrid options for data capture in REDCap and MyCap.

Participant outcome data can be collected remotely in myriad ways, including eSource data entered directly into eCRFs; EHR data [33]; lab and scan results; home sample collection; smartphones with mobile apps; ePROs (MyCap app participant surveys, REDCap participant surveys, and NIH toolbox surveys); study visits over the phone; sensors and wearable devices; blood collection devices; and web-based patient portals. A hybrid model not only allows for different types of data to be collected in different ways but also can enable the same type of data to be collected in both a traditional or decentralized fashion (Fig. 2).

The remote acquisition of data requires an understanding of technical aspects related to how data are collected, stored, and transmitted. Methods include e-sourcing, direct entry, participant capture, and data augmentation (e.g., leveraging external data sources/linkages). Data use agreements or other documentation will be necessary when intellectual property is involved.

Decentralized tools can support participants to keep track of trial activities. The OIAC19 trial recruited infant-mother pairs to evaluate the frequency of SARS-CoV-2 transmission from mothers to infants and to describe the outcomes of those infected. Mothers enrolled themselves and their infants through a REDCap link provided by a clinician or a site representative. Mothers completed patient-reported outcomes (PROs) via a survey and shared medical records for themselves and their infants. The traditional method – a lengthy, labor-intensive, fee-per-request process – requires mothers to sign a medical release allowing the trial team to request records from their health providers. The decentralized method uses the PLUTO Health Pulse app, allowing study participants to link and share both their own and their child’s data directly with the study quickly and inexpensively.

Maintaining privacy during remote research processes can pose challenges for researchers. Systems need to be rendered secure, and participants need to feel reassured that their privacy and data are safeguarded in an online environment that might include recorded video calls or survey answers subject to unintended disclosure [34]. In the Parent Sleep Education in Autism (Autism Sleep) study [24], REDCap survey data collection provided both a secure link and verified login [24]. Participants may also have trouble finding privacy within their homes; thus, the data gathered via video call or survey may be affected by this lack of privacy.

### Monitoring

Participant adherence to study protocols can also be monitored remotely [35] by a coordinating or data center. In REACT-AF [14], an assessment at trial onset identified potential risks to participants. The study team set thresholds, built dashboards, and embedded notifications directly within the electronic data capture (EDC) system to alert them to risks. The monitors can enter queries directly into the EDC to communicate with sites while leveraging the cloud-based document upload capability for source document verification when needed. Within the dashboard, a monitor can see all open unresolved queries for participants at a single site or consortium-wide with direct link to the EDC page and data field in question. Hospital records and other source documents are uploaded to the HIPAA-secure system, or an approved electronic meeting platform is leveraged for screen sharing. This system, which replaces the traditional on-site monitoring visit, saves time, but can increase sites’ document upload efforts.

### Retention/Reminders

Remote methods can be used to keep participants actively engaged in a trial, especially by providing automated reminders when study tasks are due. These can be delivered via phone calls or in-app notifications. In the Concussion Assessment, Research and Education for Kids (CARE4Kids) study [26], MyCap sends reminder notifications to participants through the application and allows for remote data entry of PROs. REDCap is also used to send email reminders to participants to complete PROs via remote REDCap surveys. A drawback to these retention methods is the potential for participants to become frustrated by or immune to frequent task reminders (e.g., daily). Offering customized frequencies for reminders is slated for future MyCap development.

### Return of Results/Return of Value

Increased emphasis has been placed in recent years on returning value to research participants, which can include return of research results. The CSSC-004^19^ Convalescent Plasma trial has employed patient-centered virtual meetings to communicate trial results and appreciation of trial participants. REACT-AF [14] has plans in place to disseminate results via teleconference.

Note, however, that a significant amount of community engagement and translation work needs to happen up-front to ensure planned content and delivery of results is meaningful to research participants and able to be interpreted by non-study team clinicians if needed. This is true of any trial, but especially those in which results may be transmitted remotely without a trained coordinator to help with explanations if there are questions. Furthermore, participants are probably less likely to ask questions about results and maybe even less likely to ask for results at all if the investigative team has not previously established a relationship with them.

**Figure 3.**
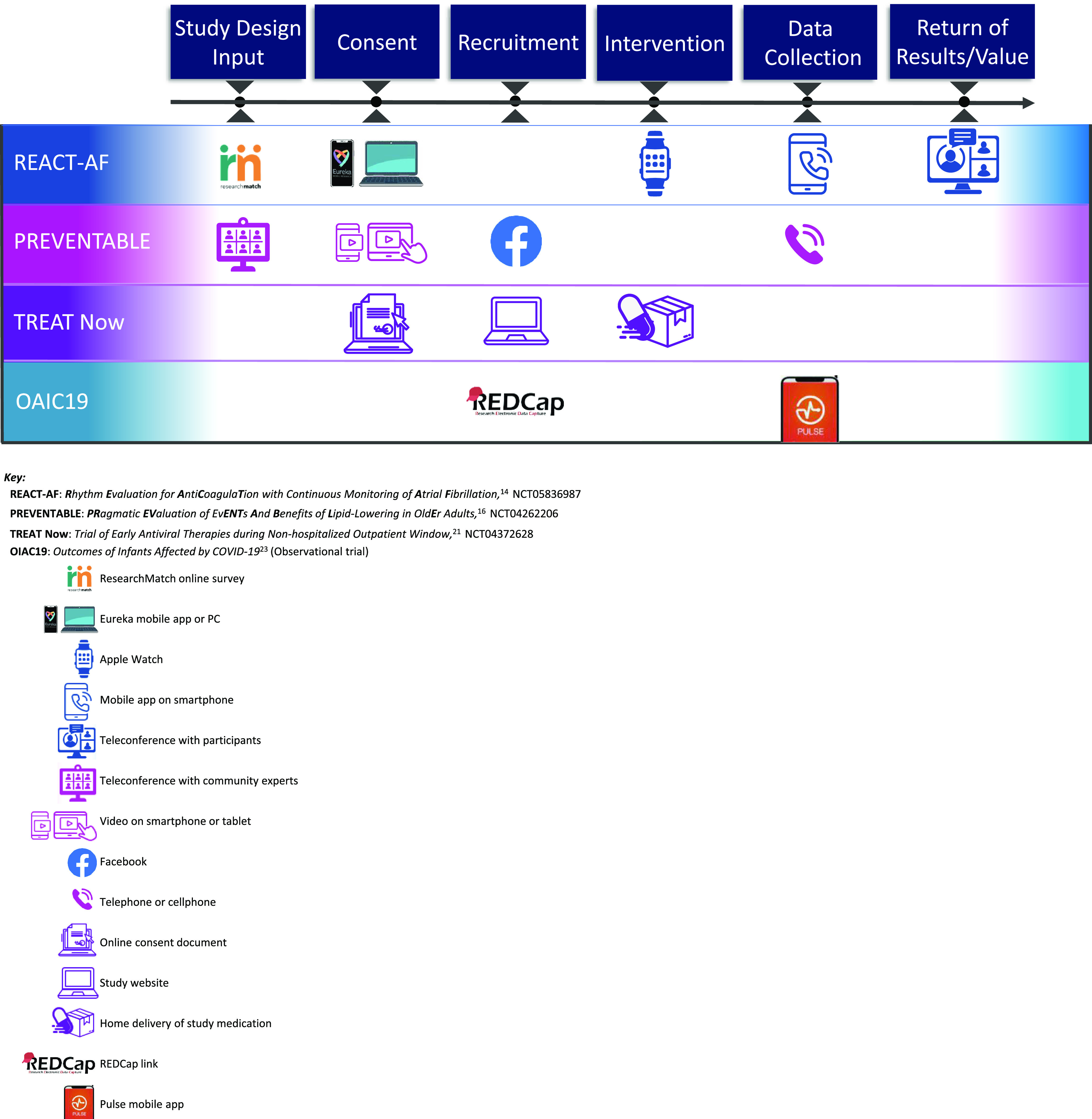
Examples of decentralized elements in Trial Innovation Network hybrid trials.

A summary of some of the specific decentralized elements used in TIN trials illustrates the hybrid nature of many of the trials (Fig. 3).

## TIN Successes and Opportunities in Decentralized Trials

All trials are unique, and that uniqueness includes opportunities for custom use and optimization of decentralized elements across and within individual study conduct phases. Based on our work with numerous trials, we have organized general areas of success and opportunity, provided below, along with specific examples gleaned from learnings in individual trials.

### Operational

An operational success example is the Autism Sleep study [24]. While initially designed for local participations at three sites, it was quickly revamped during COVID-19 to an almost fully remote trial. The redesign allowed new participants well outside the original catchment areas to take part. A Parent Portal, using a REDCap online platform for its base, was customized to enable the research team to implement multiple research functions remotely, including consent, delivery of educational interventions, survey data collection, participant notifications and reminders, automated tracking of completed tasks, and basic data analysis.

Study management and communication among sites remain essential in a decentralized trial environment and can be supported by dashboards available to all enrolling sites. In the CSSC-004^19^ Convalescent Plasma trial, decentralization was made possible by using production specifications and an innovative database platform designed to track testing standards at production locations. Tools and devices such as the Apple Watch and other FDA-approved devices can be used for trial implementation. Sponsor-provided technologies can be offered to participants, but Internet access and ability to use the devices must be considered. Technology training can be made available in multiple languages. Notably, while consumer-grade devices may be more accessible for decentralized use, they may also have less precision than medical-grade devices.

### Safety

Participant safety, critical in any trial, poses new challenges in remote settings, where investigators must rely to some extent on individual participants to self-monitor and self-report adverse outcomes. Remote monitoring procedures need careful consideration, documentation, and implementation. Because of potential risks, only some trials – for example, those with well-characterized investigational medicines or well-tested safety protocol infrastructures – may be a fit for decentralization [36].

The REACT-AF [14] study implemented a decentralized safety protocol in the form of PROs, particularly on hospitalizations. The need for additional verification of these primary endpoint events created a challenge with a technical solution: geofencing. Embedded in the Eureka mobile application is the ability to ping a participant anytime they cross a pre-defined A-GPS boundary of a hospital for a specified length of time. When the participant confirms the hospitalization, a survey is triggered in the mobile app to collect information on it. At the time of consent, participants can opt into connecting their EHR record to the app (i.e., MyChart) to allow for seamless transfer of hospital discharge summaries to the study team, further decentralizing the process. Participants can also access the traditional method of confirming their consent to the hospital by uploading the record to the HIPAA-compliant cloud EDC. In this way, safety surveillance using direct-to-participant tools in a remote setting is augmented by traditional site-based data capture and interview-based event capture.

### Inclusive Recruitment and Participation of Marginalized Populations

Low levels of clinical trial participation among marginalized populations are a well-established challenge in clinical research. Generalizability of study results is impacted when trial participation is not inclusive. Decentralized trials are uniquely positioned to reach a wide range of participants by removing geographic and time constraints associated with study participation [19,37], and early evidence suggests that online recruitment and trial conduct can reduce participation disparities [38]. However, the use of technology and home visits, while potentially helpful in lessening participant study burden, may still present challenges for participants who are resource insecure or unaccustomed to using technology independently.

Older adults in the PREVENTABLE study often struggle with the technology needed for remote trial tasks, and those with hearing or visual impairments who are unable to use the technology are not eligible to join the study. Efforts are being made in other studies to provide the special adaptations to technology that mobility-impaired participants may need. During the conceptualization of the *All of Us* program [39], community members with various “diversabilities” were invited to give feedback on aspects of program development. Those who were blind/had limited vision or were deaf/hard of hearing were engaged to provide thought leadership around the assistive technology needed to participate in the program. Lessons learned focused on intentionally designing platforms and trials with these populations in mind and included the need to provide access to web interfaces that can be read by a screen reader, and closed captioning on webpages and electronic consent documents.

Rural populations can face substantial hurdles in traveling to clinical trial sites and thus are often underrepresented in clinical research [40]. Remote technologies offer tremendous value in this context [10]. For example, the use of an online Parent Portal in the Autism Sleep Study [24] widened participant inclusion by enabling those in areas far from a research center an opportunity to participate. The PREVENTABLE [16] trial used elements of direct-to-participant telehealth, including home delivery of study medication and remote follow-up (obtained from the participant’s health records, by telephone, or a home visit) to reduce participant burden, maintain engagement, and reach diverse populations. This approach is also applicable to rare disease patients whose trial participation is disadvantaged by distance from clinical trial sites [41]. In the hybrid Cavernous Angiomas with Symptomatic Hemorrhage (CASH) trial, a staged approach of initial participant evaluation at geographically local sites was followed by airplane travel for a single day of core lab testing at the national core lab [25].

The multilingual needs of diverse trial participants offer both a challenge and an opportunity. In the Autism Sleep Study [24], more than a third (36%) of the participating children were Latino/Hispanic [24]. Online study materials were offered in both English and Spanish. However, translating the study platform and surveys was deemed logistically prohibitive. Notably, PROs may require translation, cultural adaptation, and validation to be fully accessible in a multilingual context. Because many tools that leverage technology for remote trials have yet to enable validated translations, the technology can constitute a barrier to culturally appropriate participation where survey-based outcomes reporting is required.

Lack of trust has an impact on clinical trial participation, yet creating trust without in-person, face-to-face discussion can be challenging. Researchers may help overcome misgivings by meeting potential participants within a trusted environment such as their homes, local churches, and pharmacies. However, decentralized trials are new to these populations. A failure to establish a strong relationship with communities prior to engaging in research can jeopardize the participant-trial relationship. The CSSC-004 Convalescent Plasma Trial [19] worked within four distinct Navajo communities to gain trust, by turning to local clinicians, tribal decision makers, established tribal regulatory processes, and local pharmacies, but did not achieve recruitment proportional to disease burden in the Navajo communities over the 12 initial months of the pandemic. Decentralization is not always an immediate solution to increased diversity in participation, particularly if trust with communities relevant to the study has not been established. Although the TIN has had success in incorporating community engagement into study design, building trust is a time- and effort-intensive process that may be difficult with completely remote approaches.

## Conclusion

The TIN’s early experiences with hybrid decentralized trials can be instructive to researchers considering remote approaches to multisite clinical trials, and many of the lessons learned are likely applicable to future trials, even those not fully decentralized. While some decentralized methods, such as eConsent, have proven effective, others, such as remote screening/enrollment, initial evaluation, and monitoring need more development and operational assessment before their value is known. Participants may lack digital access or skills or be uneasy about online protections. The ideal approach would give research participants options for participation, although this may place additional burden on research coordinators. Hybrid trials that include some decentralized elements, combined with traditional approaches, may be more immediately feasible and provide opportunities for increasing participant reach, streamlining processes, and cost savings. While decentralized trials may reduce geographic barriers to participation, their impact on participation of racial and ethnic minority populations has not been extensively measured. The TIN is in a unique position to learn lessons across many multisite trials and to assess decentralized operational successes and challenges. There is still work ahead to understand when and how decentralized elements should be used; however, new technologies and remote approaches show promise to support efficient, informative trials that engage participants with minimal burden.
